# Geological Survey of Kentucky

**Published:** 1853-10

**Authors:** 


### GEOLOGICAL SURVEY OF KENTUCKY.

Almost every State in the Union has had a geological survey. Kentucky, thus far, has been blind to her interests on this subject. We think we see indications of an awakening in reference to it, and suggest to our Kentucky readers to press the matter upon the attention of their Representatives in the Legislature. The measure can be consummated at the next session if its friends will make a vigorous and united effort in its behalf. It is high time the work were begun.

